# Brain structure and function in adult survivors of developmental trauma with psychosis: A systematic review

**DOI:** 10.1017/S0033291724002812

**Published:** 2025-09-15

**Authors:** Michael Bloomfield, Mustapha Modaffar, Alexander Noar, Camilla Walker, Ting-Yun Chang

**Affiliations:** 1 Translational Psychiatry Research Group, Research Department of Mental Health Neuroscience, Division of Psychiatry, UCL Institute of Mental Health, University College London, London, UK; 2 Clinical Psychopharmacology Unit, Research Department of Clinical, Educational and Health Psychology, University College London, London, UK; 3 Department of Neurology, National Hospital for Neurology & Neurosurgery, London, UK; 4 NIHR University College London Hospitals Biomedical Research Centre, London, UK; 5 The Traumatic Stress Clinic, St Pancras Hospital, Camden and Islington NHS Foundation Trust, London, UK; 6 Department of Mathematical & Physical Sciences, University College London, London, UK; 7 Department of Neuroscience, Physiology & Pharmacology, Division of Biosciences, University College London, London, UK

**Keywords:** Developmental trauma, Psychosis, Neuroimaging

## Abstract

**Background:**

Developmental trauma increases psychosis risk in adulthood and is associated with poor prognosis and treatment response. It has been proposed that developmental trauma may give rise to a distinct psychosis phenotype. Our aim was to explore this by systematically reviewing neuroimaging studies of brain structure and function in adults with psychosis diagnoses, according to whether or not they had survived developmental trauma. We registered our search protocol in PROSPERO (CRD42018105021).

**Method:**

We systematically searched literature databases for relevant studies published before May 2024. We identified 31 imaging studies (*n* = 1,761 psychosis patients, *n* = 1,775 healthy controls or healthy siblings).

**Results:**

Developmental trauma was associated with global and regional differences in gray matter; corticolimbic structural dysconnectivity; a potentiated threat detection system; dysfunction in regions associated with mentalization; and elevated striatal dopamine synthesis capacity.

**Conclusion:**

These findings warrant further research to elucidate vulnerability and resilience mechanisms for psychosis in developmental trauma survivors.

## Introduction

There is consistent evidence that developmental trauma (including maltreatment in childhood and adolescence) increases risk of psychosis (McGrath et al., 2017). People experiencing psychosis are more than twice as likely to have experienced developmental trauma (Varese et al., 2012) and 15 times more likely to have experienced childhood sexual abuse (CSA) than people without psychosis (Bebbington et al., 2004). Evidence that developmental trauma causes psychosis fulfils Bradford Hill criteria (Hill, 1965), including strong and consistent associations between trauma and psychosis (Varese et al., 2012); temporal relationships (Kelleher et al., 2013); dose effects (Duhig et al., 2015; Longden, Sampson, & Read, 2016; Schäfer & Fisher, 2011); and increased risk of conversion from at-risk states to first-episode psychosis (Brew, Doris, Shannon, & Mulholland, 2018). Developmental trauma may account for up to a third of psychosis cases (McGrath et al., 2017), and is associated with poor prognosis and treatment response (Cakir, Tasdelen Durak, Ozyildirim, Ince, & Sar, 2016; Misiak & Frydecka, 2016); the latter may be suggestive of distinct and/or additional neurobiological mechanisms underlying psychotic phenomena. Despite this, we lack a precise mechanistic understanding of how developmental trauma alters brain structure and function to give rise to psychosis. This may represent a barrier to developing more effective treatments for this patient group (Bloomfield et al., 2020).

Various brain alterations are associated with psychosis (Bloomfield, Buck, & Howes, 2016). Childhood and adolescence are sensitive periods for brain development (Goddings & Giedd, 2014), including synaptic pruning, synaptogenesis, and myelination (Miller et al., 2012). Developmental trauma can disrupt brain development to cause lasting changes in structure and function (McCrory, Gerin, & Viding, 2017; Teicher, Samson, Anderson, & Ohashi, 2016). These include reduced volume of the hippocampus and anterior cingulate cortex, altered fiber tract density in the corpus callosum, and altered sensory systems (Teicher et al., 2016). Animal research using stress paradigms indicates potential processes underlying these alterations include aberrant dendritic arborization and inhibition of neurogenesis (Czéh et al., 2001; Magariños, McEwen, Flügge, & Fuchs, 1996). In parallel, several neurocognitive domains of direct relevance to psychotic symptomatology are particularly sensitive to the effects of developmental trauma, including aberrant amygdalar responsivity during threat processing, striatal reward processing dysfunction, impaired frontal emotion regulation, and executive control (McCrory et al., 2017). Therefore, it is likely that developmental trauma results in changes to brain structure and function that can give rise to psychosis (Read, Fosse, Moskowitz, & Perry, 2014). It is, therefore, imperative to understand the underlying neurobiological mechanisms accounting for this.

It has been proposed that adult survivors of developmental trauma with psychosis represent a distinct clinical phenotype from those who have not experienced developmental trauma, underlined by differences in brain structure and function (Read et al., 2014). Such phenotypes have also been proposed clinically using subgroups such as ‘traumatic psychosis’, ‘neurodevelopmental psychosis’, and ‘psychotic PTSD’ to describe distinct manifestations of psychosis (Bloomfield et al., 2021; Stevens, Spencer, & Turkington, 2017). Given the implications of understanding underlying mechanisms for developing targeted treatments, we sought to address whether there are differences in brain structure and function within patients with psychosis according to whether they have or have not survived developmental trauma. Our hypothesis was that within people experiencing psychosis, there are structural and functional brain differences between those with or without a developmental trauma history. We tested this by systematically reviewing the neuroimaging literature of people experiencing psychosis with and without a history of developmental trauma.

## Methods and materials

### Search strategy

We preregistered this systematic review with PROSPERO (CRD42018105021) and followed the Preferred Reporting Items for Systematic Reviews and Meta-Analyses (PRISMA) (Moher et al., 2009). Preliminary search was conducted on 3rd January 2019, using PubMed and MedLine (M.A.P.B., M.M), and a definitive search was conducted on 19th May 2024 using PubMed, MedLine, Web of Science, and PsychINFO via Ovid (M.A.P.B., T-Y. C.). For both searches, we used a combination of AND/OR operators using the search string: (child* OR adolesc* OR develop*) AND (schizophrenia OR psychosis OR ‘psychotic’) AND (abus* OR maltreat* OR trauma* OR advers* OR neglect) AND (‘gray matter’ OR ‘magnetic resonance imaging’ OR ‘connectivity’ OR ‘salience network’ OR ‘resting state’ OR ‘default mode’ OR ‘white matter’ OR ‘DTI’ OR ‘PET’ OR ‘SPECT’ OR ‘Computed Tomography’). The numerous possible iterations were also input individually into the search engines, and additional results that did not appear in systematic searches were noted. A librarian was consulted on the search strategy.

### Selection criteria

We sought to address whether there were differences in brain structure and function between patients with psychosis who have survived developmental trauma compared to patients with psychosis who have not. We assessed studies against predetermined criteria for inclusion in the review: original studies in English up until 19th May 2024 of adult participants between the ages of 18–65; studies comparing or measuring brain structure and function in participants with psychosis, either in two groups (with or without developmental trauma) or along a gradient of developmental trauma exposure, via neuroimaging techniques specified in the search (MRI, DTI, PET, SPECT, or CT); studies were included of participants across the natural history of psychosis, including clinical high-risk states, first-episode or chronic stages; both medicated and unmedicated patients; and individuals at familial high risk. High-risk patients who had not experienced psychosis were included as there is evidence that trauma induces vulnerability to psychosis across the spectrum of severity (Bechdolf et al., 2010). Developmental trauma was frequently defined using the Childhood Trauma Questionnaire Short Form, but there was substantial heterogeneity with other studies using alternative questionnaires, including the Childhood Life Events Questionnaire, the Early Trauma Inventory, the Traumatic Experiences Check-List, and the Childhood Experiences of Care and Abuse Questionnaire. Peer victimization or neighborhood-level exposures, such as crime, were not considered, as these are not reliably measured in many neuroimaging studies. Exclusion criteria were: studies without measures of brain structure and/or function; studies only including healthy participants; and studies involving both underage and adult participants that did not distinguish between age groups. In studies involving patient groups selected for a particular trait (e.g. history of violence), these data were disregarded, as they are not representative of the general patient population.

Screening of abstracts and full text was done by two researchers, APN and CW, using Covidence. The same two researchers performed the data extraction using a pre-made data extraction chart in Covidence. Any disagreements were then discussed with the lead author, MAPB, where a final decision was made.

### Quality and risk of bias assessment

The Newcastle–Ottawa quality assessment scale was used to assess methodological quality and risk of bias (Wells et al., 2011). Rating results are presented below in Table 6.

## Results

**Figure 1.**
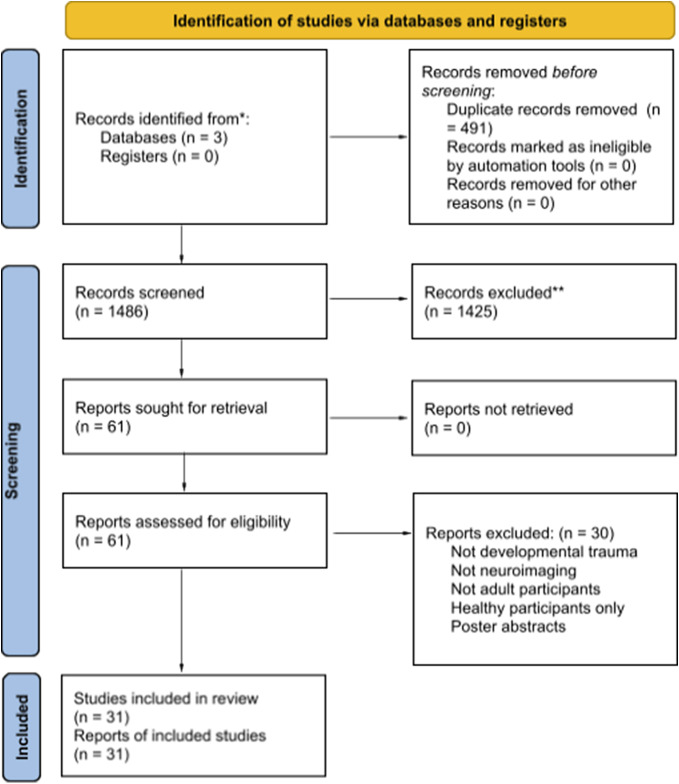
PRISMA 2020 flow diagram for new systematic reviews, which included searches of databases and registers only. *Consider, if feasible to do so, reporting the number of records identified from each database or register searched (rather than the total number across all databases/registers). **If automation tools were used, indicate how many records were excluded by a human and how many were excluded by automation tools.

The selection process is presented in Figure 1. We identified 31 suitable published studies. Thirteen studies used structural magnetic resonance imaging (MRI), five used diffusion tensor imaging (DTI), eleven used functional MRI (fMRI), one used positron emission tomography (PET), and one study used MRI and fMRI. Nineteen of the included studies met criteria for good quality (Aas et al., 2017; Asmal et al., 2019; Domen et al., 2019; Egerton et al., 2016; Frissen, van Os, Peeters, Gronenschild, & Marcelis, 2018; Habets, Marcelis, Gronenschild, Drukker, & Van Os, 2011; Hoy et al., 2012; Kumari et al., 2013; Kumari et al., 2014; Neilson et al., 2017; Peeters, et al., 2015a,b; Quidé et al., 2017a,b; Dauvermann et al., 2021; Quidé, Girshkin, Watkeys, Carr, & Green, 2021; King et al., 2022; Costello et al., 2023; Xie et al., 2023). All but three studies assessed substance use in patients (Aas et al., 2012; Aas et al., 2017; Costello et al., 2023). Out of 31 studies, 20 excluded participants with current and past substance dependence (Allen et al., 2018; Asmal et al., 2019; Barker et al., 2016a,b; Cancel et al., 2017; Dauvermann et al., 2021; King et al., 2022; Kumari et al., 2013; Kumari et al., 2014; Poletti et al., 2015; Quidé et al., 2021; Quidé et al., 2017a,b; Quidé, O’Reilly, Watkeys, Carr, & Green, 2018; Ruby, Rothman, Corcoran, Goetz, & Malaspina, 2017; Xie et al., 2023) and seven studies covaried for drug use (Domen et al., 2019; Egerton et al., 2016; Frissen et al., 2018; Habets et al., 2011; Neilson et al., 2017; Peeters et al., 2015a,b).

### Structural imaging

#### Cortical structure

All of three studies investigating cortical structure found that compared to people with psychosis who had not been exposed to developmental trauma (Table 1), developmental trauma is associated with reduced thickness or surface area of the cortex (Barker et al., 2016a; Habets et al., 2011 ; Neilson et al., 2017). In one high-quality study (*n* = 88), people with psychosis exhibited a negative relationship between cortical thickness and developmental trauma, whereas healthy siblings exhibited a positive relationship (Habets et al., 2011). Similarly, another high-quality study (*n* = 99) found that in patients with psychosis, developmental trauma reduces the cortical thickness of the right temporal lobe, whereas in healthy controls, an opposite pattern was observed (Neilson et al., 2017). In both studies (Habets et al., 2011; Neilson et al., 2017), there was no relationship between antipsychotic drug exposure and cortical thickness, suggesting that pharmacotherapy was unlikely to be driving the effects. The third study (*n* = 145) of participants at high familial risk of psychosis used social services involvement as a proxy marker of developmental trauma, finding reduced bilateral hemispheric surface area in the developmental trauma survivor group relative to individuals not exposed to trauma (Barker et al., 2016a). This study found no difference in cortical thickness between the two groups.

**Table 1.** Cortical and regional structure findings using magnetic resonance imaging

Study	Participants (*n*)	Medication status	Method of medication control	Results
Cortical thickness and surface area				
Habets et al. (2011)	HC = 87, psychotic disorder = 88 (SCZ = 62, SZA = 9, SZP = 4, brief psychotic disorder = 2, psychotic disorder not otherwise specified = 11), 98 healthy siblings	73/88 patients medicated	Estimated lifetime dopamine antagonist use using haloperidol equivalents	*Whole brain* Reduced cortical thickness associated with trauma severity in SCZ
				Negative association between cortical thickness and trauma exposure in SCZ/HC Positive correlation between cortical thickness and high trauma exposure in healthy siblings
Barker et al. (2016a)	fHR = 145	Unmedicated	–	*Whole brain* Reduced bilateral hemisphere surface area in trauma survivors (*n* = 48) versus no trauma (*n* = 97)
				No effect on bilateral cortical thickness
Neilson et al. (2017)	HC = 41; SCZ = 38; BD = 20	Medicated	Estimated lifetime dopamine antagonist using chlorpromazine equivalents	*Region of interest* Positive association between cortical thickness of right temporal lobe and DT in HC
				Negative association between cortical thickness of right temporal lobe and DT in SCZ/BD
Regional volumes				
Ruby et al. (2017)	SCZ, SZA = 18	Medicated	Participants not matched	*Whole brain and region of interest* Negative correlation between trauma severity and WB volume in SCZ/SZA
				Positive correlation between trauma severity and amygdala:WB volume ratio (due to increased amygdala volumes)
Hoy et al. (2012)	FEP = 21 (10 = SCZ, 3 = BPAD, 2 = psychotic depression, 6 = other psychotic disorder)	Medicated (short-term exposure)	Time from first clinical presentation to MRI scan as a proxy measure for duration of antipsychotic exposure	*Region of interest: Amygdala and hippocampus* Reduced left hippocampus volume in trauma survivors with FEP (*n* = 16) versus no trauma (*n* = 5).
				Reduced total and right amygdala volume in trauma survivors with FEP (*n* = 16) versus no trauma (*n* = 5).
Aas et al., (2012)	HC = 63, FEP = 83	75/83 patients medicated	Estimated lifetime dopamine antagonist use using chlorpromazine equivalents	*Region of interest: Amygdala and hippocampus* Negative association between trauma severity (in terms of polyvictimization) and bilateral amygdala volume in FEP.
Barker et al. (2016b)	fHR = 140	Unmedicated	–	*Whole brain* Reduced right hippocampus and left amygdala volumes in trauma survivors (*n* = 43) versus no trauma (*n* = 97)
Benedetti et al. (2011)	HC = 20, SCZ = 20	Medicated	Estimated lifetime dopamine antagonist use using chlorpromazine equivalents	*Region of interest: Amygdala, hippocampus, anterior cingulate cortex, prefrontal cortex* Increased GMV of anterior cingulate and prefrontal cortex in trauma survivors with SCZ (*n* = 10) versus no trauma (*n* = 10).
				Reduced GMV of anterior cingulate and prefrontal cortex in no trauma SCZ group (*n* = 10), but trauma severity associated with increased GMV in anterior cingulate and prefrontal cortex in trauma survivor SCZ group (*n* = 10).
Sheffield et al. (2013)	HC = 26, psychotic disorder = 60 (26 SCZ, 17 SZA, 17 BPAD)	Medicated	Participants not matched	*Whole brain* Negative association between CSA severity and global GMV.
				Negative association between CSA severity and bilateral prefrontal cortex GMV in P + CSA versus P-CSA.
Cancel et al. (2015)	HC = 30, SCZ = 21	Medicated	Estimated lifetime dopamine antagonist use using chlorpromazine equivalents	*Whole brain* Negative association between emotional neglect severity and global GMV in SCZ.
				Negative association between emotional neglect severity and right dorsolateral prefrontal cortex GMV in SCZ.
Poletti et al. (2016)	HC = 136, BPAD = 206 SCZ = 96	Medicated	Estimated lifetime dopamine antagonist use using chlorpromazine equivalents	*Whole brain* Reduced orbitofrontal cortex and thalamus GMV in BPAD/SCZ + DT (*n* = 151) versus HC. No significant difference when comparing BPAD/SCZ − DT (*n* = 151) to HC.
Frissen et al. (2018)	HC = 87, psychotic disorder = 89, 95 siblings	Medicated	Estimated lifetime dopamine antagonist use using haloperidol equivalents	*Whole brain* Negative association between trauma severity and global GMV in psychotic disorders.
Kumari et al. (2013)	HC = 15; SCZ = 15 (Disregarded groups: violent ASPD = 13; violent SCZ = 13).	Medicated	Estimated lifetime dopamine antagonist use using chlorpromazine equivalents	*Whole brain* Negative association between DT and GMV of left inferior frontal region in all individuals
				Negative association between DT and GMV in the left middle frontal and precentral gyri in HC and SCZ
				*Region of interest: cerebellum, temporal lobe, lateral ventricles, caudate nucleus, putamen, thalamus, hippocampus, amygdala, prefrontal and occipito-parietal regions* Negative association between DT and occipito-parietal, prefrontal, and hippocampal volume in both SCZ and HC
Kumari et al. (2014)	HC = 15; SCZ = 15 (Disregarded groups: violent ASPD = 14; violent SCZ = 15)	Medicated	Estimated lifetime dopamine antagonist use using chlorpromazine equivalents	*Region of interest: anterior cingulate cortex* Negative association between DT and anterior cingulate volumes in all participants
				No group differences when DT ratings were covaried for

Abbreviations: BPAD, Bipolar affective disorder; CSA, Childhood sexual abuse; DT, Developmental trauma; FEP, First-episode psychosis; fHR, Familial high-risk; GMV, Gray matter volume; HC, Healthy controls; MRI, Magnetic resonance imaging; P + DT, Psychosis and developmental trauma; P − DT, Psychosis without developmental trauma; SCZ, Schizophrenia; SZA, Schizoaffective disorder; SZP, Schizophreniform disorder.

#### Global gray matter volume

There is high-quality evidence for the association between developmental trauma and reduced gray matter volume (GMV) across any brain region (Table 1) (Benedetti et al., 2011; Cancel et al., 2015; Frissen et al., 2018; Kumari et al., 2013; Kumari et al., 2014; Poletti et al., 2016; Ruby et al., 2017; Sheffield, Williams, Woodward, & Heckers, 2013).

#### Frontal gray matter volume

Findings of reduced regional GMV in people with psychosis who experienced developmental trauma were reported in four out of five studies (Table 1) (Benedetti et al., 2011; Cancel et al., 2015; Poletti et al., 2016; Sheffield et al., 2013). One study had a large (*n* = 302) sample of medicated participants with chronic (> 10 years) bipolar disorder or schizophrenia (Poletti et al., 2016). Compared to patients with psychosis with low levels of developmental trauma (P − DT), patients with psychosis who have survived developmental trauma (P + DT) showed reduced GMV in the orbitofrontal cortex (OFC), when compared to healthy controls (Poletti et al., 2016). There was some evidence that specific types of trauma were associated with region-specific alterations in brain structure. For example, exposure to CSA was associated with reduced prefrontal GMV (Sheffield et al., 2013), while exposure to emotional neglect was associated with reduced GMV in the dorsolateral prefrontal cortex (DLPFC) which in turn mediated the severity of disorganization symptoms (Cancel et al., 2015). Another high-quality study reported a negative association between ratings of psychosocial deprivation and GMV in the left inferior frontal region and left middle frontal precentral gyri (Kumari et al., 2013).

#### Medial temporal and subcortical gray matter volume

Four out of five studies found that P + DT were associated with smaller medial temporal volumes, specifically reduced amygdala and/or hippocampus volumes (Table 1) (Aas et al., 2012; Barker et al., 2016a; Hoy et al., 2012; Kumari et al., 2013). There was high-quality evidence of negative relationships between trauma exposure and the volumes of these structures from two studies (Aas et al., 2012; Kumari et al., 2013). Importantly, one high-quality study (Hoy et al., 2012) (*n* = 21) of medicated FEP patients reported that 24% of patients met PTSD criteria (using the Posttraumatic Diagnostic Scale) (Foa, Riggs, Dancu, & Rothbaum, 1993) in relation to their developmental trauma experiences.

For subcortical structures, in one large study (*n* = 302) (Poletti et al., 2016), reduced thalamic GMV was found in P + DT relative to healthy trauma survivors (Table 1). No studies were found measuring or reporting alterations in striatal structures.

#### White matter

In three out of five studies, there is high-quality evidence that within patients with psychosis, developmental trauma is associated with reduced white matter microstructure measured as reduced functional anisotropy (FA) and increased mean diffusivity (MD) (Table 2) (Asmal et al., 2019; Domen et al., 2019; Poletti et al., 2015). One study (*n* = 83) found that connectivity was inversely related to the degree of developmental trauma in white matter tracts linking gray matter structures that also exhibit volumetric deficits described above (Poletti et al., 2015), including the corpus callosum, cingulum, corona radiata, inferior longitudinal fasciculus, and thalamic radiation. Importantly, there was also prospective evidence of inverse relationship between level of trauma and mean FA observed over time in the patient group (Domen et al., 2019), which was not observed in other groups and remained significant when controlling for medication. Together, both of these studies suggest a dose–response effect of trauma exposure on the extent of white matter alterations. There was also some evidence for particular types of trauma being associated with patterns of structural connectivity from one study (*n* = 54) of minimally medicated FEP patients (< 4 weeks cumulative lifetime exposure to dopamine antagonists) (Asmal et al., 2019). In that high-quality study, sexual abuse was associated with reduced FA in the inferior fronto-occipital fasciculus, inferior longitudinal fasciculus, and the superior longitudinal fasciculus, whilst emotional neglect was associated with increased FA in the right superior longitudinal fasciculus, relative to patients without experiences of developmental trauma. Two studies found that although both patients with psychosis and individuals with a history of DT showed reduced FA in similar areas, there were no differences between patients with psychosis, as well as DT, and those without (Costello et al., 2023; Xie et al., 2023).

**Table 2.** Structural connectivity findings using diffusion tensor imaging

Author	Participants (*n*)	Medication status	Method of medication control	Results
Poletti et al. (2015)	SCZ = 83	Medicated	Estimated lifetime antipsychotic use (method of estimation not stated)	Negative association between trauma severity and FA in: corpus callosum, left cingulum, left corona radiata, bilateral superior longitudinal fasciculus, left inferior longitudinal fasciculus, left thalamic radiation.
				Positive association between trauma severity and FA in: right superior/inferior fronto-occipital fasciculus, right inferior longitudinal fasciculus, right corticospinal tract, anterior/posterior right thalamic radiation, major/minor forceps, right cingulum, bilateral corpus callosum, right anterior/posterior/superior corona radiata.
Asmal et al. (2019)	HC = 51, FEP = 54	Medicated	Exclusion if lifetime exposure > 4 weeks	Reduced FA in left inferior longitudinal fasciculus, bilateral superior longitudinal fasciculus, right inferior fronto-occipital fasciculus in P + DT (*n* = 16) versus HC + DT (*n* = 13).
				Reduced FA in right inferior fronto-occipital fasciculus, right inferior longitudinal fasciculus, right forceps major in P + CSA (*n* = 13) versus P-CSA (*n* = 41).
				Increased FA in right superior longitudinal fasciculus in P + EN (*n* = 17) versus P-EN (*n* = 37).
Domen et al. (2019)	Controls = 49 (of which 11 have MDD), Psychotic disorder = 55 (SCZ = 33, SZA = 16, psychotic disorder not specified = 6), 55 siblings	Medicated	Estimated lifetime dopamine antagonist use using haloperidol equivalents	In P + DT versus siblings + DT and controls + DT: negative association between trauma severity and mean FA on follow-up at 3 years.
Costello et al. (2023)	HC = 129, SCZ = 37	Medicated	Estimated lifetime dopamine antagonist use using chlorpromazine equivalents	SCZ showed FA reductions in the corpus callosum and corona radiata compared to HC.
				Irrespective of a diagnosis, high DT levels were related to FA reductions in frontolimbic and frontoparietal regions compared to those with none/low levels of DT.
				No significant interaction between diagnosis and high levels of DT was found.
Xie et al. (2023)	HC = 206, FEP = 103	48/103 medicated	Estimated lifetime dopamine antagonist use using olanzapine equivalents	Compared with the HCs group, the FEP group showed significantly lower FA in several white matter bundles (left anterior thalamic radiation, left inferior frontal-occipital fasciculus, left cingulum, forceps major, and forceps minor).
				The mean FA value in these white matter bundles was inversely related to the level of DT.

Abbreviations: CSA, Childhood sexual abuse; DT, Developmental trauma; DTI, Diffusion tensor imaging; EN, Emotional neglect; FA, Fractional anisotropy; FEP, First-episode psychosis; HC, Healthy controls; MD, Mean diffusivity; MDD, Major depressive disorder; P + DT, Psychosis and developmental trauma; P − DT, Psychosis without developmental trauma; SCZ, Schizophrenia; SZA, Schizoaffective disorder.

### Functional imaging

#### Cerebral perfusion

One study in UHR (*n* = 77) measured resting state cerebral perfusion of the hippocampus, basal ganglia, and midbrain using arterial spin labelling (Allen et al., 2018), relative to healthy volunteers (Table 3). Participants on antipsychotic medication were excluded from the final analyses. There was a positive relationship between level of developmental trauma (CTQ score) and resting state cerebral blood flow in the right hippocampus/subiculum and left parahippocampal gyrus in the UHR group. The whole brain analysis found a negative association between developmental trauma and perfusion in a cluster encompassing the left IFG and superior/medial PFC in the UHR group.

**Table 3.** Arterial spin labelling imaging findings

Study	Participants (*n*)	Medication status	Method of medication control	Results
Resting cerebral blood flow (rCBF)				
Allen et al. (2018)	HC = 25, UHR = 77	8/77 patients medicated	Exclusion of medicated participants	*Whole brain* Negative association between trauma severity and resting cerebral blood flow of cluster spanning left inferior frontal gyrus and superior/medial prefrontal cortex in UHR versus HC
				*Region of interest: hippocampus, basal ganglia* Positive association between trauma severity and resting cerebral blood flow of right hippocampus/subiculum, left parahippocampal gyrus in UHR versus HC

Abbreviations**:** BPD, Borderline personality disorder, BPAD, Bipolar affective disorder, DT, Developmental trauma, HC, Healthy controls, P + DT, Psychosis and developmental trauma, P − DT, Psychosis without developmental trauma, SCZ, Schizophrenia, SZA, Schizoaffective disorder, UHR, Ultra-high risk.

#### Resting state

Two high-quality fMRI studies investigated the effects of developmental trauma on functional connectivity in the same sample (*n* = 228) of patients with schizophrenia (Peeters, et al., 2015a,b). While there is no significant association between developmental trauma and functional connectivity between regions of the default mode network (Peeters et al., 2015b), there is a positive association between trauma exposure and nucleus accumbens–lentiform nucleus connectivity (Peeters et al., 2013). A further study showed increased connectivity between the medial prefrontal cortex and the cerebellum in patients with schizophrenia with high levels of trauma in comparison to those with low levels of trauma (Dauvermann et al., 2021).

#### Emotional processing

Four of the fMRI studies investigated emotional processing using face matching (Table 4) (Aas et al., 2017; Benedetti et al., 2011; Cancel et al., 2017; Quidé et al., 2021). A large, high-quality study (*n* = 101) of mostly medicated patients with schizophrenia or bipolar spectrum diagnoses found task-induced hyperactivation when differentiating between responses to negative and positive emotional valence in middle temporal and lateral occipital cortex, which was associated with trauma exposure (Aas et al., 2017). One functional connectivity analysis in a smaller sample (*n* = 21) of medicated schizophrenia patients found that CSA was dose-dependently associated with hypoconnectivity between the amygdala–left posterior cingulate cortex/precuneus and amygdala–right calcarine sulcus (Cancel et al., 2017). One study showed that in response to a stressor (an MRI session), patients with high levels of DT showed decreased activation in bilateral temporo-parietal-insular junctions, right middle cingulum, right pre–postcentral gyrus, and left cerebella lobules IV–VI, while there was increased activation in patients with low levels of DT (Quidé et al., 2021). Finally, Benedetti and colleagues (Benedetti et al., 2011) investigated the amygdala, hippocampus, ACC, and PFC as ROIs in a small sample (*n* = 20) of medicated patients with schizophrenia using fearful and angry faces. Comparing high and low trauma groups, trauma-specific ACC and PFC hyperactivation was found relative to both patient and control groups without trauma, which remained significant when controlling for medication. Collectively, within patients with psychosis, developmental trauma is associated with alterations in emotional processing. MRI findings have recently extended to investigate default mode network hubs in an affective theory of mind (ToM) task in medicated patients with schizophrenia or schizoaffective disorder (*n* = 47) (Quidé et al., 2017b). This high-quality study found a relationship between trauma exposure and posterior cingulate hyperactivation in patients, suggesting that developmental trauma may result in functional brain changes contributing to abnormal self-oriented mental imagery. In the whole brain analysis, trauma exposure was associated with superior frontal hyperactivation and temporo-parietal hypoactivation.

**Table 4.** Functional magnetic resonance imaging findings

Study	Participants (*n*)	Medication status	Method of medication control	Results
Emotional processing				
Benedetti et al. (2011)	HC = 20, SCZ = 20	Medicated	Estimated lifetime dopamine antagonist use using chlorpromazine equivalents	*Region of interest: Amygdala, hippocampus, anterior cingulate cortex, prefrontal cortex* In SCZ/HC + DT (*n =* 20) versus SCZ/HC − DT (*n* = 20), reduced activation of amygdala, hippocampus.
				In SCZ/HC + DT (*n =* 20) versus SCZ/HC − DT (*n =* 20), increased activation in anterior cingulate cortex, prefrontal cortex.
				In SCZ + DT (*n =* 10) versus HC + DT (*n =* 10), decreased activation in prefrontal cortex, anterior cingulate cortex.
				In SCZ + DT (*n =* 10) versus HC + DT (*n =* 10), increased activation of right amygdala, hippocampus.
Aas et al. (2017)	SCZ/BPAD spectrum = 101	Medicated (76/101)	Daily dose of antipsychotics (method of conversion not stated)	*Whole brain* Increased activation in right angular gyrus, supramarginal gyrus, middle temporal gyrus, lateral occipital cortex in P+ high DT group (*n* = 48) versus P+ low DT (*n* = 53) for negative > positive faces.
				In psychosis, developmental physical abuse associated with increased activity during differentiation of neg. > pos. Faces in bilateral superior temporal gyrus, angular gyrus, Heschl’s gyrus, insula, pre/postcentral gyrus, right precentral gyrus, putamen, central opercular cortex, intracalcarine gyrus.
Cancel et al. (2017)	HC = 25, SCZ = 21 (overlap with (Cancel et al., 2015)	Medicated	Estimated lifetime dopamine antagonist use using chlorpromazine equivalents	*Region of interest: Amygdala seed region* Reduced functional connectivity associated with developmental sexual abuse in psychosis between amygdala–left posterior cingulate/precuneus, amygdala–right calcarine sulcus.
				In psychosis, developmental physical neglect associated with reduced connectivity between amygdala–left precuneus
Quidé et al. (2021)	HC = 34, SCZ/SZA = 40, BPAD = 35	Medicated	Estimated lifetime dopamine antagonist use using chlorpromazine equivalents	*Whole brain* The severity of DT moderated the relationship between cortisol reactivity and brain activation in the bilateral temporo-parieto-insular junctions, right middle cingulum, right pre/postcentral gyri, left cerebellum, and right lingual gyrus, differently depending on the clinical group.
				SCZ patients showed a blunted cortisol response. Those with high levels of DT showed reduced activation in bilateral temporo-parietal-insular junctions, middle cingulum, and left cerebellar lobules, whereas those with low levels of DT showed increased activation of these areas.
				HC with high levels of DT showed decreased activation in these areas in response to increasing cortisol reactivity, whereas those with low levels of DT showed increased activation in these areas.
Response inhibition				
Quidé et al. (2018)	HC = 53, SCZ, SZA, BPAD = 112	Medicated	Estimated lifetime dopamine antagonist use using chlorpromazine equivalents	*Region of interest: bilateral inferior frontal gyrus, right dorsolateral prefrontal cortex, right supplementary motor area, right inferior parietal lobule, dorsal anterior cingulate cortex* Trauma survivors (*n* = 71) versus no trauma (*n* = 41) in psychotic disorders group show 9% increase in activation in left inferior frontal gyrus during response inhibition, mediated by general symptom severity.
Working memory				
Quidé et al. (2017a)	HC = 47, SCZ, SZA, BPAD = 92	Medicated	Estimated lifetime dopamine antagonist use using chlorpromazine equivalents	*Region of interest: bilateral dorsolateral prefrontal cortex, medial prefrontal cortex, posterior cingulate, dorsal anterior cingulate* Increased activation of bilateral cuneus, calcarine sulcus, lingual gyrus in P + DT versus P − DT, HC + DT.
				Increased activation in SCZ + DT versus SCZ − DT in left superior temporal gyrus, pre/postcentral gyrus, post. Insula, Rolandic operculum, Heschl’s gyrus, superior temporal pole.
Theory of mind				
Quidé et al. (2017b)	SCZ, SZA = 47	Medicated	Estimated lifetime dopamine antagonist use using chlorpromazine equivalents	*Whole brain* Increased activation in right medial superior frontal gyrus, supplementary motor area (posterior to dorsomedial prefrontal cortex in P + DT versus P − DT
				Reduced activation in right Rolandic operculum, superior temporal gyrus, superior marginal gyrus, posterior insula in P + DT versus P − DT
				*Region of interest: bilateral temporo-parietal junction, dorsomedial prefrontal cortex, posterior cingulate cortex/precuneus* Increased activation in both posterior cingulate cortex/precuneus for P + DT versus P − DT
Dauvermann et al. (2021)	HC = 116, SCZ/SZA = 57	Medicated	Estimated lifetime dopamine antagonist use using chlorpromazine equivalents available for 41/57 patients	Region of interest: medial prefrontal cortex, right lateral parietal lobe, left lateral parietal lobe, and posterior cingulate cortex Widespread reductions in functional connectivity in patients versus controls, including between the left/right parietal lobe and multiple other regions, including the parietal operculum bilaterally.
				Increased DT scores were associated with increased prefrontal-cerebellar connectivity in patients.
				DT-associated differences in default mode network connectivity also predicted variation in behavioral measures of ToM in both patients and controls.
King et al. (2022)	HC = 176, SCZ/SZA = 51	Not described	Not described	*Region of interest: medial prefrontal cortex, right lateral parietal, left lateral parietal, and posterior cingulate cortex.* Patients showed significantly increased default mode network connectivity compared to healthy participants between each of the four seeds of the default mode network and with other clusters in the brain.
				Across the entire sample, higher levels of IL–6 predicted increased connectivity between the mPFC and regions encompassing the cerebellum.
				IL–6 mediated the association between physical neglect and weaker suppression of the posterior cingulate cortex (PCC) default mode network seed–left precentral and postcentral gyrus connectivity during ToM performance.
Functional connectivity				
Peeters et al. (2015a)	HC = 72; PD = 73; nonpsychotic siblings of PD = 83	64/73 medicated	Estimated lifetime dopamine antagonist use using haloperidol equivalents	*Region of interest: left inferior parietal lobule; left precuneus; right medial prefrontal cortex* No association between DT and default mode network functional connectivity
Peeters et al. (2015b)	HC = 59; PD = 63; nonpsychotic siblings of PD = 73	Medicated	Estimated lifetime dopamine antagonist use using haloperidol equivalents	*Region of interest: middle frontal gyrus (MFG); orbitofrontal cortex (OFC); lentiform nucleus (LN); middle cingulate cortex(MCC)* Positive association between DT and nucleus accumbens–lentiform nucleus connectivity

#### Executive processing

Response inhibition and working memory have been investigated for which both studies were significant. An ROI analysis of a large sample (*n* = 112) during a Go/No-Go Flanker task showed that developmental trauma was associated with hyperactivation of the left inferior frontal gyrus (IFG) (Table 4) (Quidé et al., 2018). Task-induced IFG hyperactivation was associated with general symptom severity within P + DT, but also in P − DT. A separate study (*n* = 92) investigated default mode network hubs during visuo-spatial working memory processing (Quidé et al., 2017a). Trauma exposure was associated with increases in activation of the left inferior parietal lobule, without a behavioral difference in working memory performance between groups, possibly reflecting reduced cortical efficiency and/or compensatory mechanisms.

### Molecular imaging

We identified one high-quality PET study (Egerton et al., 2016) of striatal dopamine synthesis capacity in UHR (*n* = 47) reporting a measure of developmental trauma (Table 5) (Bifulco, Brown, & Harris, 1994). Developmental trauma was associated with elevated striatal dopamine synthesis capacity, particularly in the associative functional striatal subdivision (that is dorsal caudate and putamen), compared to low exposure. However, there was no significant difference in dopamine function between the ultra-high-risk participants who survived developmental trauma and controls with traumatic experiences.

**Table 5.** Molecular imaging using 18-F DOPA PET

Author	Participants (*n*)	Medication status	Method of medication control	Results
Egerton et al. (2016)	HC = 20, UHR = 47	2/47 patients medicated	Exclusion of medicated participants	*Volume of interest: striatum* Positive correlation between total CAARMS (53) and dopamine synthesis capacity in UHR versus HC for sensorimotor striatum, but not associative or whole striatum.
				No significant differences in dopamine function in UHR + DT versus HC + DT

Abbreviations: CAARMS, Comprehensive assessment of at-risk mental states; DT, Developmental trauma; PET, Positron emission tomography; UHR, Ultra-high risk.

**Table 6.** Quality and risk of bias assessment results using the Newcastle–Ottawa assessment scale

Legend	
◯	Circle indicates that the paper meets the criteria
✕	Cross indicates that the paper does not meet the criteria
*Totalscore*	A total score of 7 or more for the case control and cohort studies, and of 6 or more for the cross-sectional studies, is indicative of ‘good’ quality and bias control.

Case–control studies										
Study	Selection				Comparability		Exposure			Total
	Adequate case definition	Representativeness of cases	Selection of controls	Definition of controls	Comparability of cases and controls on basis of the design or analysis		Ascertainment of exposure	Same method of ascertainment for cases and controls	Nonresponse rate	Total/9
Habets et al. (2011)	◯	◯	◯	◯	◯	◯	✕	◯	✕	**7**
Barker et al. (2016a)	◯	◯	✕	✕	◯	✕	✕	◯	✕	**4**
Barker et al. (2016b)	◯	◯	✕	✕	◯	✕	✕	◯	✕	**4**
Neilson et al. (2017)	◯	◯	◯	◯	◯	◯	✕	◯	✕	**7**
Aas et al. (2012)	◯	◯	✕	✕	◯	◯	✕	✕	✕	**4**
Sheffield et al. (2013)	◯	✕	◯	✕	◯	✕	✕	◯	✕	**4**
Cancel et al. (2015)	◯	✕	✕	◯	◯	◯	✕	◯	✕	**5**
Poletti et al. (2016)	◯	◯	✕	✕	◯	◯	✕	◯	✕	**5**
Frissen et al. (2018)	◯	◯	◯	◯	◯	◯	✕	◯	✕	**7**
Kumari et al. (2013)	◯	◯	◯	◯	✕	◯	◯	◯	✕	**7**
Kumari et al. (2014)	◯	◯	◯	◯	✕	◯	◯	◯	✕	**7**
Asmal et al. (2019)	◯	◯	◯	◯	◯	◯	✕	◯	✕	**7**
Domen et al. (2019)	◯	◯	◯	◯	◯	◯	✕	◯	✕	**7**
Allen et al. (2018)	◯	◯	✕	✕	◯	◯	✕	✕	✕	**4**
Cancel et al. (2017)	◯	✕	✕	◯	◯	◯	✕	◯	✕	**5**
Quidé et al. (2018)	◯	◯	◯	◯	✕	◯	✕	◯	✕	**6**
Quidé et al. (2017a)	◯	◯	◯	◯	◯	◯	✕	◯	✕	**7**
Peeters et al. (2015a)	◯	◯	◯	◯	◯	◯	✕	◯	✕	**7**
Peeters et al. (2015b)	◯	◯	◯	◯	◯	◯	✕	◯	✕	**7**
Egerton et al. (2016)	◯	◯	◯	◯	◯	◯	✕	◯	✕	**7**
Benedetti et al. (2011)	◯	✕	✕	◯	◯	◯	✕	◯	✕	**5**
Costello et al. (2023)	◯	◯	◯	◯	◯	◯	✕	◯	✕	**7**
Dauvermann et al. (2021)	◯	◯	◯	◯	◯	◯	✕	◯	✕	**7**
King et al. (2022)	◯	◯	◯	◯	✕	◯	✕	◯	✕	**6**
Quidé et al. (2021)	◯	◯	◯	◯	✕	◯	✕	◯	✕	**6**
Xie et al., (2023)	◯	◯	◯	◯	◯	◯	✕	◯	✕	**7**

Cross-sectional studies								
Study	Selection			Comparability		Outcome		Total
	Representativeness of the sample	Nonrespondents	Ascertainment of the exposure (valid measure)	Subjects in different outcome groups are comparable, based on study design or analysis. Confounding factors are controlled		Assessment of the outcome (blinded?)	Statistical test	Total/7
Ruby et al. (2017)	◯	✕	◯	◯	✕	◯	◯	**5**
Hoy et al. (2012)	◯	✕	◯	◯	◯	◯	◯	**6**
Poletti et al. (2015)	✕	✕	◯	◯	◯	◯	◯	**5**
Aas et al. (2017)	◯	✕	◯	◯	◯	◯	◯	**6**
Quidé et al. (2017b)	◯	✕	◯	◯	◯	◯	◯	**6**

## Discussion

Our neuroimaging review investigated brain structure and function in survivors of developmental trauma with psychosis across the whole psychotic spectrum including ultra-high-risk individuals. We have found evidence in support of our hypothesis that there are differences in brain structure and function in adults with psychosis who have or have not survived developmental trauma. These included small global, frontal, and subcortical volumes, low corticolimbic connectivity, and alterations in brain function during cognitive processing. Whilst the majority of studies were cross-sectional, there was high-quality prospective evidence of putative trauma-related effects alongside dose effects of trauma exposure on changes in brain structure (Domen et al., 2019) which may suggest causation.

There are several possible interpretations for our findings that there appear to be neuroimaging differences between people with psychosis who report having or having not survived developmental trauma, which are not mutually exclusive. First, trauma-induced changes in brain structure and function may induce vulnerability to psychosis. This interpretation would be consistent with findings from other studies that developmental trauma exposure is associated with changes in structure and function in circuits that are implicated in psychosis (Bloomfield et al., 2016; Teicher & Samson, 2016; Xie et al., 2023). Moreover, additive interactions with genetic and other environmental factors could possibly also lead to increased illness severity. This is because global brain volume reductions are observed in schizophrenia (Giedd et al., 1999) and in those at genetic risk (Cooper, Barker, Radua, Fusar-Poli, & Lawrie, 2014). Accumulated trauma-induced changes (Liberzon & Sripada, 2007) may have an additive effect on such volume reductions (Ruby et al., 2017). This could represent neurobiological pathways to poorer prognosis (Cakir et al., 2016; Misiak & Frydecka, 2016). Thus, trauma-induced alterations in brain structure and function may underlie worsened psychosis symptomatology following trauma (Duhig et al., 2015). Findings of reduced cortical thickness and hippocampal may also provide underlying neurobiological changes to match to a distinct traumatogenic phenotype. However, further research identifying the clinical factors and response to treatment is required to explore this possibility further.

However, it remains unknown which trauma-induced changes in brain structure and function may be associated with resilience. Therefore, an alternative explanation is that brain changes associated with trauma may be adaptations and not necessarily pathological in otherwise healthy individuals. It is also possible that the findings reviewed here are not related to trauma per se but may be due to pre-existing (intrinsic) differences in brain structure and function, and/or variance in etiology of psychotic disorders. Intrinsic brain variations preceding trauma exposure may serve as risk factors underlying the development of psychotic symptoms following the experience of a traumatic event. Prospective, longitudinal studies are needed to elucidate possible phenotypes associated the development of psychosis following trauma exposure. Furthermore, due to the clinical overlap between PTSD and psychosis, these findings may be driven by PTSD. Importantly, PTSD symptoms are often overlooked in patients with psychosis, and studies of patients with psychosis often do not report PTSD symptoms (Zammit et al., 2018). Future studies are needed to measure the relationships between developmental trauma, psychopathology, resilience, and alterations in neurobiology to investigate this further.

Findings of divergent brain alterations in patients reporting developmental trauma, according to the presence or absence of psychosis, are striking (Domen et al., 2019; Habets et al., 2011). One possible interpretation of these findings is that they may reflect resilience and/or compensatory mechanisms, and further work is needed to understand underlying processes. Whilst speculative, possible resilience and vulnerability factors may include susceptibility to stress-induced changes in dendritic arborization and neuronal migration (Lyall et al., 2015). Taken together, it remains unknown if potential differences in brain structure and function are due to an additive effect of developmental trauma on psychosis symptomatology or whether psychosis following developmental trauma represents a distinct clinical phenotype and further investigation, including genetic and longitudinal research, is needed to address this.

This study has implications for understanding the neurocognitive processes underlying how developmental trauma may cause psychosis, including through executive and threat processing. In terms of executive function, the PFC is one of the final cortical structures to mature (Huttenlocher, 1990), rendering it especially vulnerable to stressors during development (McCrory et al., 2017; Teicher & Samson, 2016), and there is evidence that neglect-induced reductions in DLPFC GMV are associated with disorganization symptoms in patients (Cancel et al., 2015). Hyperactivation in executive function domains of working memory and response inhibition may reflect attempted compensatory mechanisms necessary to maintain similar levels of behavioral performance on such tasks (Quidé et al., 2018; Quidé, et al., 2017a). Since the PFC is critical for executive function and emotion regulation, dysfunction in regions where there are structural alterations associated with developmental trauma may underlie cognitive impairments (Benedetti et al., 2011; Dannlowski et al., 2012; Üçok et al., 2015). Moreover, there is evidence that the PFC is involved in fear extinction (Fullana et al., 2018). Deficits in PFC GMV associated with psychosis patients with developmental trauma may, thus, be an explanation for the maintenance of paranoia.

Findings of smaller hippocampal volumes (Aas et al., 2012; Barker et al., 2016a; Hoy et al., 2012; Ruby et al., 2017) are in keeping with studies in adolescent survivors of developmental trauma without psychosis (Opel et al., 2014; Teicher et al., 2018) and adults with PTSD or a dissociative disorder (Logue et al., 2018; Pitman et al., 2012). Glucocorticoid exposure impairs neuronal growth (Czéh et al., 2001), and the hippocampus is highly sensitive to excessive glucocorticoids (Sapolsky, 1996). FEP patients who have survived developmental trauma showed reduced levels of brain-derived neurotrophic factor (BDNF) combined with elevated levels of cortisol, which predicted smaller hippocampal volumes (Mondelli et al., 2011). Hippocampal atrophy is the most consistently reported structural finding in PTSD (Pitman et al., 2012) whereby reduced hippocampal volume may predispose individuals to PTSD and there is also evidence that trauma exposure further reduces hippocampal volumes (Pitman et al., 2012). Furthermore, a recent study found evidence for hippocampal sensitive periods in early life, during which traumatic experiences were associated with reduced hippocampal volume (Humphreys et al., 2019). It is, therefore, possible that hippocampal dysfunction could give rise to psychotic experiences. These findings are also consistent with evidence that PTSD symptoms may be involved in the relationship between developmental trauma and psychosis (Bloomfield et al., 2021). However, given that stress results in changes to hippocampal structure in several psychiatric disorders (Geuze, Vermetten, & Bremner, 2005), it is possible that these findings are not specific to psychosis and further research is needed to address the potential role in reduced hippocampal volume in the pathophysiology of psychosis associated with developmental trauma.

Paranoia is a key symptom of psychosis causing high levels of distress (Freeman, Garety, Kuipers, Fowler, & Bebbington, 2002) and is associated with developmental trauma (Read & Argyle, 1999). We found evidence that psychosis in patients reporting developmental trauma is associated small amygdalar volumes (Aas et al., 2012; Barker et al., 2016a; Hoy et al., 2012; Kumari et al., 2013) and hyperactivation during threat processing (Aas et al., 2017; Benedetti et al., 2011). One explanation is that putative trauma-induced structural changes during sensitive periods occur alongside sensitization of threat processing (Humphreys et al., 2019; Teicher et al., 2016). These findings are in keeping with findings that emotional dysregulation is involved in the link between developmental trauma and psychosis (Bloomfield et al., 2021). Potentiated threat detection may develop in adverse environments via attentional biases, as survivors exhibit faster identification of negative valence emotional stimuli than nonmaltreated controls (Masten et al., 2008). Small ACC volumes may result in impaired top-down amygdalar inhibition which would hyper-potentiate threat detection, removing the ‘brakes’ on an already accelerated system (Humphreys et al., 2019). Reduced activation in the temporo-parieto-insular junctions could also contribute to poorer emotional/threat processing further compounding these issues (Quidé et al., 2021). Animal models suggest that this may arise from impaired GABA-based inhibition during fear learning (Piantadosi & Floresco, 2014). This interpretation is consistent with PTSD models that describe a hyper-responsive amygdala alongside a hypo-responsive PFC to threat (Liberzon & Sripada, 2007).

### Strengths and limitations

Key strengths of our review include preregistration and the synthesis of multimodal neuroimaging literature across phases of psychosis, adding to previous reviews (Cancel, Dallel, Zine, El-Hage, & Fakra, 2019; Thomas et al., 2023). However, this review is not without its limitations. These relate to the existing neuroimaging data and to the directionality of causal relationships between traumata, the brain, and psychotic symptoms.

In terms of limitations of the field, there is currently a lack of studies that distinguish between different experiences of developmental trauma in terms of type, severity, and age of exposure. The grouping together of different experiences of trauma into a single entity is a limitation of the current literature study given that different experiences of trauma at different ages are likely to be associated with different effects on the development of brain structure and function. The heterogeneity and small number of published studies for each of the MRI methods is a further limitation. This also limits our ability to detect publication bias across our review. In terms of causality, the cross-sectional nature of the body of research presented here precludes causal inferences of directionality of dynamic changes in brain structure and function assumed to be associated with developmental trauma. We cannot exclude the possibility of reverse causation whereby putative differences in brain structure and function are not caused by trauma exposure, but rather increase risk that a child will be maltreated (Kelleher et al., 2013). There are also a range of possible confounds in this field. For example, given that trauma incidence is higher in more socioeconomically deprived communities (Elliot, 2016), contextual social factors may be responsible for some of the effects. Retrospective assessment of trauma is a recurrent limitation (Okeke, Wilkinson, & Roberts, 2017) and we cannot exclude the possibility that recall bias is influencing our results. However, patients with psychosis under-report and minimize, rather than over-report and exaggerate trauma severity (Church, Andreassen, Lorentzen, Melle, & Aas, 2017). The majority of studies also employ small samples and were heterogeneous in the types of developmental trauma reported. Whilst most studies accounted for medication dosage by regression analysis or lifetime exposure, it remains possible that our findings could be due to effects of long-term antipsychotics (Fusar-Poli et al., 2013). As we did not restrict our research question to survivors of developmental trauma with a diagnosis of schizophrenia, it is possible that our inclusion of studies of patients with other clinical presentations limits the inferences that can be made from our study. On the other hand, our study did not include patients with schizotypy, which is associated with similar brain alterations to schizophrenia (Kirschner et al., 2022), and there is some evidence for an impact of childhood trauma on gray matter alterations in schizotypy (Quidé et al., 2024). Finally, as most studies did not report the presence of PTSD symptoms, we cannot exclude the possibility that comorbid PTSD accounts for some of our findings. Future work is, therefore, urgently needed to address these considerations.

## Conclusion

Patients with psychosis who have survived developmental trauma may exhibit alterations in brain structure and function compared to those without histories of trauma. There is some overlap with findings in posttraumatic stress disorder which may be pertinent to understanding the neurocognitive basis of psychotic symptoms following developmental trauma. Further research is urgently needed to precisely elucidate neurocognitive mechanisms giving rise to psychosis following developmental trauma. In parallel, we must also elucidate mechanisms of resilience. Understanding these processes may facilitate the development of more effective treatments for trauma survivors to prevent fully established psychosis and aid those experiencing psychosis in achieving remission and recovery.

## References

[r1] Aas, M., Kauppi, K., Brandt, C. L., Tesli, M., Kaufmann, T., Steen, N. E., … Melle, I. (2017). Childhood trauma is associated with increased brain responses to emotionally negative as compared with positive faces in patients with psychotic disorders. Psychological Medicine, 47(4), 669–679. 27834153 10.1017/S0033291716002762

[r2] Aas, M., Navari, S., Gibbs, A., Mondelli, V., Fisher, H. L., Morgan, C., … Dazzan, P. (2012). Is there a link between childhood trauma, cognition, and amygdala and hippocampus volume in first-episode psychosis? Schizophrenia Research, 137(1–3), 73–79. 22353995 10.1016/j.schres.2012.01.035

[r3] Allen, P., Azis, M., Modinos, G., Bossong, M. G., Bonoldi, I., Samson, C., … McGuire, P. (2018). Increased resting hippocampal and basal ganglia perfusion in people at ultra high risk for psychosis: Replication in a second cohort. Schizophrenia Bulletin, 44(6), 1323–1331. 29294102 10.1093/schbul/sbx169 PMC6192497

[r4] Asmal, L., Kilian, S., du Plessis, S., Scheffler, F., Chiliza, B., Fouche, J. P., … Emsley, R. (2019). Childhood trauma associated white matter abnormalities in first-episode schizophrenia. Schizophrenia Bulletin, 45(2), 369–376. 29860345 10.1093/schbul/sby062 PMC6403087

[r5] Barker, V., Bois, C., Johnstone, E. C., Owens, D. G. C., Whalley, H. C., McIntosh, A. M., & Lawrie, S. M. (2016a). Childhood adversity and cortical thickness and surface area in a population at familial high risk of schizophrenia. Psychological Medicine, 46(4), 891–896. 26654172 10.1017/S0033291715002585

[r6] Barker, V., Bois, C., Neilson, E., Johnstone, E. C., Owens, D. G. C., Whalley, H. C., … Lawrie, S. M. (2016b). Childhood adversity and hippocampal and amygdala volumes in a population at familial high risk of schizophrenia. Schizophrenia Research, 175(1–3), 42–47. 27179666 10.1016/j.schres.2016.04.028

[r7] Bebbington, P. E., Bhugra, D., Brugha, T., Singleton, N., Farrell, M., Jenkins, R., … Meltzer, H. (2004). Psychosis, victimisation and childhood disadvantage: Evidence from the second British National Survey of psychiatric morbidity. The British Journal of Psychiatry, 185(3), 220–226. 15339826 10.1192/bjp.185.3.220

[r8] Bechdolf, A., Thompson, A., Nelson, B., Cotton, S., Simmons, M. B., Amminger, G. P., … Yung, A. R. (2010). Experience of trauma and conversion to psychosis in an ultra-high-risk (prodromal) group. Acta Psychiatrica Scandinavica, 121(5), 377–384. 20199494 10.1111/j.1600-0447.2010.01542.x

[r9] Benedetti, F., Radaelli, D., Poletti, S., Falini, A., Cavallaro, R., Dallaspezia, S., … Smeraldi, E. (2011). Emotional reactivity in chronic schizophrenia: Structural and functional brain correlates and the influence of adverse childhood experiences. Psychological Medicine, 41(3), 509–519. 20529416 10.1017/S0033291710001108

[r10] Bifulco, A., Brown, G. W., & Harris, T. O. (1994). Childhood experience of care and abuse (CECA): A retrospective interview measure. Journal of Child Psychology and Psychiatry, 35(8), 1419–1435. 7868637 10.1111/j.1469-7610.1994.tb01284.x

[r11] Bloomfield, M. A., Buck, S. C., & Howes, O. D. (2016). Schizophrenia: Inorganic no more. Lancet Psychiatry, 3(7), 600–602. 10.1016/S2215-0366(16)30096-7. 27371980

[r12] Bloomfield, M. A. P., Yusuf, F., Srinivasan, R., Kelleher, I., Bell, V., & Pitman, A. (2020). Trauma-informed care for adult survivors of developmental trauma with psychotic and dissociative symptoms: A systematic review of intervention studies. Lancet Psychiatry, 7(5), 449–462. 10.1016/S2215-0366(20)30041-9. 32004444

[r13] Bloomfield, M. A., Chang, T., Woodl, M. J., Lyons, L. M., Cheng, Z., Bauer-Staeb, C., … Lewis, G. (2021). Psychological processes mediating the association between developmental trauma and specific psychotic symptoms in adults: A systematic review and meta-analysis. World Psychiatry, 20(1), 107–123. 33432756 10.1002/wps.20841 PMC7801841

[r14] Brew, B., Doris, M., Shannon, C., & Mulholland, C. (2018). What impact does trauma have on the at-risk mental state? A systematic literature review. Early Intervention in Psychiatry, 12(2), 115–124. 28560861 10.1111/eip.12453

[r15] Cakir, S., Tasdelen Durak, R., Ozyildirim, I., Ince, E., & Sar, V. (2016). Childhood trauma and treatment outcome in bipolar disorder. Journal of Trauma & Dissociation, 17(4), 397–409. 26683845 10.1080/15299732.2015.1132489

[r16] Cancel, A., Comte, M., Boutet, C., Schneider, F. C., Rousseau, P. F., Boukezzi, S., … Fakra, E. (2017). Childhood trauma and emotional processing circuits in schizophrenia: A functional connectivity study. Schizophrenia Research, 184, 69–72. 27979699 10.1016/j.schres.2016.12.003

[r17] Cancel, A., Comte, M., Truillet, R., Boukezzi, S., Rousseau, P. F., Zendjidjian, X. Y., … Fakra, E. (2015). Childhood neglect predicts disorganization in schizophrenia through grey matter decrease in dorsolateral prefrontal cortex. Acta Psychiatrica Scandinavica, 132(4), 244–256. 26038817 10.1111/acps.12455

[r18] Cancel, A., Dallel, S., Zine, A., El-Hage, W., & Fakra, E. (2019). Understanding the link between childhood trauma and schizophrenia: A systematic review of neuroimaging studies. Neuroscience & Biobehavioral Reviews, 107, 492–504. 31163206 10.1016/j.neubiorev.2019.05.024

[r19] Church, C., Andreassen, O. A., Lorentzen, S., Melle, I., & Aas, M. (2017). Childhood trauma and minimization/denial in people with and without a severe mental disorder. Frontiers in Psychology, 8, 1276. 28883800 10.3389/fpsyg.2017.01276 PMC5573805

[r20] Cooper, D., Barker, V., Radua, J., Fusar-Poli, P., & Lawrie, S. M. (2014). Multimodal voxel-based meta-analysis of structural and functional magnetic resonance imaging studies in those at elevated genetic risk of developing schizophrenia. Psychiatry Research: Neuroimaging, 221(1), 69–77. 10.1016/j.pscychresns.2013.07.008 24239093

[r21] Costello, L., Dauvermann, M. R., Tronchin, G., Holleran, L., Mothersill, D., Rokita, K. I., … Cannon, D. M. (2023). Childhood trauma is associated with altered white matter microstructural organization in schizophrenia. Psychiatry Research: Neuroimaging, 330, 111616. 36827958 10.1016/j.pscychresns.2023.111616

[r22] Czéh, B., Michaelis, T., Watanabe, T., Frahm, J., De Biurrun, G., Van Kampen, M., … Fuchs, E. (2001). Stress-induced changes in cerebral metabolites, hippocampal volume, and cell proliferation are prevented by antidepressant treatment with tianeptine. Proceedings of the National Academy of Sciences, 98(22), 12796–12801. 10.1073/pnas.211427898 PMC60133 11675510

[r23] Dannlowski, U., Stuhrmann, A., Beutelmann, V., Zwanzger, P., Lenzen, T., Grotegerd, D., … Kugel, H. (2012). Limbic scars: Long-term consequences of childhood maltreatment revealed by functional and structural magnetic resonance imaging. Biological Psychiatry, 71(4), 286–293. 22112927 10.1016/j.biopsych.2011.10.021

[r24] Dauvermann, M. R., Mothersill, D., Rokita, K. I., King, S., Holleran, L., Kane, R., … Donohoe, G. (2021). Changes in default-mode network associated with childhood trauma in schizophrenia. Schizophrenia Bulletin, 47(5), 1482–1494. 33823040 10.1093/schbul/sbab025 PMC8379545

[r26] Domen, P., Michielse, S., Peeters, S., Viechtbauer, W., van Os, J., & Marcelis, M. (2019). Childhood trauma-and cannabis-associated microstructural white matter changes in patients with psychotic disorder: A longitudinal family-based diffusion imaging study. Psychological Medicine, 49(4), 628–638. 29807550 10.1017/S0033291718001320

[r27] Duhig, M., Patterson, S., Connell, M., Foley, S., Capra, C., Dark, F., … Scott, J. (2015). The prevalence and correlates of childhood trauma in patients with early psychosis. Australian & New Zealand Journal of Psychiatry, 49(7), 651–659. 25722463 10.1177/0004867415575379

[r28] Egerton, A., Valmaggia, L. R., Howes, O. D., Day, F., Chaddock, C. A., Allen, P., … McGuire, P. (2016). Adversity in childhood linked to elevated striatal dopamine function in adulthood. Schizophrenia Research, 176(2–3), 171–176. 27344984 10.1016/j.schres.2016.06.005 PMC5147458

[r29] Elliot, I. (2016). Poverty and mental health: A review to inform the Joseph Rowntree Foundation’s anti-poverty strategy. Mental Health Foundation.

[r30] Foa, E. B., Riggs, D. S., Dancu, C. V., & Rothbaum, B. O. (1993). Reliability and validity of a brief instrument for assessing post-traumatic stress disorder. Journal of Traumatic Stress, 6, 459–474.

[r31] Freeman, D., Garety, P. A., Kuipers, E., Fowler, D., & Bebbington, P. E. (2002). A cognitive model of persecutory delusions. British Journal of Clinical Psychology, 41(4), 331–347. 12437789 10.1348/014466502760387461

[r32] Frissen, A., van Os, J., Peeters, S., Gronenschild, E., & Marcelis, M. (2018). Evidence that reduced gray matter volume in psychotic disorder is associated with exposure to environmental risk factors. Psychiatry Research: Neuroimaging, 271, 100–110. 29174764 10.1016/j.pscychresns.2017.11.004

[r33] Fullana, M. A., Albajes-Eizagirre, A., Soriano-Mas, C., Vervliet, B., Cardoner, N., Benet, O., … Harrison, B. J. (2018). Fear extinction in the human brain: A meta-analysis of fMRI studies in healthy participants. Neuroscience & Biobehavioral Reviews, 88, 16–25. 29530516 10.1016/j.neubiorev.2018.03.002

[r34] Fusar-Poli, P., Smieskova, R., Kempton, M. J., Ho, B. C., Andreasen, N. C., & Borgwardt, S. (2013). Progressive brain changes in schizophrenia related to antipsychotic treatment? A meta-analysis of longitudinal MRI studies. Neuroscience & Biobehavioral Reviews, 37(8), 1680–1691. 23769814 10.1016/j.neubiorev.2013.06.001 PMC3964856

[r35] Geuze, E. E. J. D., Vermetten, E., & Bremner, J. D. (2005). MR-based in vivo hippocampal volumetrics: 2. Findings in neuropsychiatric disorders. Molecular Psychiatry, 10(2), 160–184. 15356639 10.1038/sj.mp.4001579

[r36] Giedd, J. N., Jeffries, N. O., Blumenthal, J., Castellanos, F. X., Vaituzis, A. C., Fernandez, T., … Rapoport, J. L. (1999). Childhood-onset schizophrenia: Progressive brain changes during adolescence. Biological Psychiatry, 46(7), 892–898. 10509172 10.1016/s0006-3223(99)00072-4

[r37] Goddings, A., & Giedd, J. N. (2014). Structural brain development during childhood and adolescence. In The cognitive neurosciences (pp. 15–22).

[r38] Habets, P., Marcelis, M., Gronenschild, E., Drukker, M., & Van Os, J. (2011). Reduced cortical thickness as an outcome of differential sensitivity to environmental risks in schizophrenia. Biological Psychiatry, 69(5), 487–494. 20951979 10.1016/j.biopsych.2010.08.010

[r40] Hill, A. B. (1965). The environment and disease: Association or causation? Journal of the Royal Society of Medicine, 58, 295–300. 10.1177/003591576505800503 PMC1898525 14283879

[r41] Hoy, K., Barrett, S., Shannon, C., Campbell, C., Watson, D., Rushe, T., … Mulholland, C. (2012). Childhood trauma and hippocampal and amygdalar volumes in first-episode psychosis. Schizophrenia Bulletin, 38(6), 1162–1169. 21799213 10.1093/schbul/sbr085 PMC3494041

[r42] Humphreys, K. L., King, L. S., Sacchet, M. D., Camacho, M. C., Colich, N. L., Ordaz, S. J., … Gotlib, I. H. (2019). Evidence for a sensitive period in the effects of early life stress on hippocampal volume. Developmental Science, 22(3), e12775. 30471167 10.1111/desc.12775 PMC6469988

[r43] Huttenlocher, P. R. (1990). Morphometric study of human cerebral cortex development. Neuropsychologia, 28(6), 517–527. 2203993 10.1016/0028-3932(90)90031-i

[r44] Kelleher, I., Keeley, H., Corcoran, P., Ramsay, H., Wasserman, C., Carli, V., … Cannon, M. (2013). Childhood trauma and psychosis in a prospective cohort study: Cause, effect, and directionality. American Journal of Psychiatry, 170(7), 734–741. 23599019 10.1176/appi.ajp.2012.12091169

[r45] Kirschner, M., Hodzic-Santor, B., Antoniades, M., Nenadic, I., Kircher, T., Krug, A., … Modinos, G. (2022). Cortical and subcortical neuroanatomical signatures of schizotypy in 3004 individuals assessed in a worldwide ENIGMA study. Molecular Psychiatry, 27(2), 1167–1176. 34707236 10.1038/s41380-021-01359-9 PMC9054674

[r46] King, S., Mothersill, D., Holleran, L., Patlola, S., McManus, R., Kenyon, M., … Donohoe, G. (2022). Childhood trauma, IL-6 and weaker suppression of the default mode network (DMN) during theory of mind (ToM) performance in schizophrenia. Brain, Behavior, & Immunity-Health, 26, 100540. 10.1016/j.bbih.2022.100540 PMC9640308 36388137

[r48] Kumari, V., Gudjonsson, G. H., Raghuvanshi, S., Barkataki, I., Taylor, P., Sumich, A., … Das, M. (2013). Reduced thalamic volume in men with antisocial personality disorder or schizophrenia and a history of serious violence and childhood abuse. European Psychiatry, 28(4), 225–234. 22944337 10.1016/j.eurpsy.2012.03.002

[r49] Kumari, V., Uddin, S., Premkumar, P., Young, S., Gudjonsson, G. H., Raghuvanshi, S., … Das, M. (2014). Lower anterior cingulate volume in seriously violent men with antisocial personality disorder or schizophrenia and a history of childhood abuse. Australian & New Zealand Journal of Psychiatry, 48(2), 153–161. 24234836 10.1177/0004867413512690

[r50] Liberzon, I., & Sripada, C. S. (2007). The functional neuroanatomy of PTSD: A critical review. Progress in Brain Research, 167, 151–169. 10.1016/S0079-6123(07)67011-3 18037013

[r51] Logue, M. W., van Rooij, S. J., Dennis, E. L., Davis, S. L., Hayes, J. P., Stevens, J. S., … Morey, R. A. (2018). Smaller hippocampal volume in posttraumatic stress disorder: A multisite ENIGMA-PGC study: Subcortical volumetry results from posttraumatic stress disorder consortia. Biological Psychiatry, 83(3), 244–253. 29217296 10.1016/j.biopsych.2017.09.006 PMC5951719

[r52] Longden, E., Sampson, M., & Read, J. (2016). Childhood adversity and psychosis: Generalised or specific effects? Epidemiology and Psychiatric Sciences, 25(4), 349–359. 26156083 10.1017/S204579601500044X PMC7137611

[r53] Lyall, A. E., Shi, F., Geng, X., Woolson, S., Li, G., Wang, L., … Gilmore, J. H. (2015). Dynamic development of regional cortical thickness and surface area in early childhood. Cerebral Cortex, 25(8), 2204–2212. 24591525 10.1093/cercor/bhu027 PMC4506327

[r54] Magariños, A. M., McEwen, B. S., Flügge, G., & Fuchs, E. (1996). Chronic psychosocial stress causes apical dendritic atrophy of hippocampal CA3 pyramidal neurons in subordinate tree shrews. Journal of Neuroscience, 16(10), 3534–3540. 8627386 10.1523/JNEUROSCI.16-10-03534.1996 PMC6579123

[r55] Masten, C. L., Guyer, A. E., Hodgdon, H. B., McClure, E. B., Charney, D. S., Ernst, M., … Monk, C. S. (2008). Recognition of facial emotions among maltreated children with high rates of post-traumatic stress disorder. Child Abuse & Neglect, 32(1), 139–153. 18155144 10.1016/j.chiabu.2007.09.006 PMC2268025

[r56] McCrory, E. J., Gerin, M. I., & Viding, E. (2017). Childhood maltreatment, latent vulnerability and the shift to preventative psychiatry-the contribution of functional brain imaging. Journal of Child Psychology and Psychiatry, 58(4), 338–357. 28295339 10.1111/jcpp.12713 PMC6849838

[r57] McGrath, J. J., McLaughlin, K. A., Saha, S., Aguilar-Gaxiola, S., Al-Hamzawi, A., Alonso, J., … Kessler, R. C. (2017). The association between childhood adversities and subsequent first onset of psychotic experiences: A cross-national analysis of 23 998 respondents from 17 countries. Psychological Medicine, 47(7), 1230–1245. 28065209 10.1017/S0033291716003263 PMC5590103

[r58] Miller, D. J., Duka, T., Stimpson, C. D., Schapiro, S. J., Baze, W. B., McArthur, M. J., … Sherwood, C. C. (2012). Prolonged myelination in human neocortical evolution. Proceedings of the National Academy of Sciences, 109(41), 16480–16485. 10.1073/pnas.1117943109 PMC3478650 23012402

[r59] Misiak, B., & Frydecka, D. (2016). A history of childhood trauma and response to treatment with antipsychotics in first-episode schizophrenia patients: Preliminary results. The Journal of Nervous and Mental Disease, 204(10), 787–792. 27441460 10.1097/NMD.0000000000000567

[r60] Moher, D., Liberati, A., Tetzlaff, J., Altman, D. G., & PRISMA Group. (2009). Preferred reporting items for systematic reviews and meta-analyses: The PRISMA statement. Annals of Internal Medicine, 151(4), 264–269. 19622511 10.7326/0003-4819-151-4-200908180-00135

[r61] Mondelli, V., Cattaneo, A., Murri, M. B., Di Forti, M., Handley, R., Hepgul, N., … Pariante, C. M. (2011). Stress and inflammation reduce brain-derived neurotrophic factor expression in first-episode psychosis: A pathway to smaller hippocampal volume. The Journal of Clinical Psychiatry, 72(12), 20080. 10.4088/JCP.10m06745 PMC4082665 21672499

[r62] Neilson, E., Bois, C., Gibson, J., Duff, B., Watson, A., Roberts, N., … Lawrie, S. M. (2017). Effects of environmental risks and polygenic loading for schizophrenia on cortical thickness. Schizophrenia Research, 184, 128–136. 27989645 10.1016/j.schres.2016.12.011

[r63] Okeke, N. L., Wilkinson, A. V., & Roberts, R. E. (2017). The stability of retrospective child sexual abuse reports and its association with problem avoidance. Journal of Child Sexual Abuse, 26(6), 677–691. 28569610 10.1080/10538712.2017.1307892

[r64] Opel, N., Redlich, R., Zwanzger, P., Grotegerd, D., Arolt, V., Heindel, W., … Dannlowski, U. (2014). Hippocampal atrophy in major depression: A function of childhood maltreatment rather than diagnosis? Neuropsychopharmacology, 39(12), 2723–2731. 24924799 10.1038/npp.2014.145 PMC4200502

[r65] Peeters, S. C., van de Ven, V., Gronenschild, E. H. M., Patel, A. X., Habets, P., Goebel, R., … Genetic Risk and Outcome of Psychosis (GROUP). (2015b). Default mode network connectivity as a function of familial and environmental risk for psychotic disorder. PLoS One, 10(3), e0120030. 25790002 10.1371/journal.pone.0120030 PMC4366233

[r66] Peeters, S., Van De Ven, V., Habets, P., Goebel, R., Van Os, J., & Marcelis, M. (2013). Childhood trauma and functional connectivity in psychotic disorder. Schizophrenia Bulletin Conference: 14th International Congress on Schizophrenia Research, ICOSR 2013 Orlando, FL United States Conference Publication: (var pagings) 39 (SUPPL 1) (pp S167), 2013 Date of Publication: May 2013, 39(Suppl. 1), S167. 10.1093/schbul/sbt011.

[r67] Peeters, S. C. T., Gronenschild, E. H. B. M., Van De Ven, V., Habets, P., Goebel, R., Van Os, J., & Marcelis, M. (2015a). Altered mesocorticolimbic functional connectivity in psychotic disorder: An analysis of proxy genetic and environmental effects. Psychological Medicine, 45(10), 2157–2169. 25804977 10.1017/S0033291715000161

[r68] Piantadosi, P. T., & Floresco, S. B. (2014). Prefrontal cortical GABA transmission modulates discrimination and latent inhibition of conditioned fear: Relevance for schizophrenia. Neuropsychopharmacology, 39(10), 2473–2484. 10.1038/npp.2014.99. 24784549 PMC4138759

[r69] Pitman, R. K., Rasmusson, A. M., Koenen, K. C., Shin, L. M., Orr, S. P., Gilbertson, M. W., … Liberzon, I. (2012). Biological studies of post-traumatic stress disorder. Nature Reviews Neuroscience, 13(11), 769–787. 23047775 10.1038/nrn3339 PMC4951157

[r70] Poletti, S., Mazza, E., Bollettini, I., Locatelli, C., Cavallaro, R., Smeraldi, E., & Benedetti, F. (2015). Adverse childhood experiences influence white matter microstructure in patients with schizophrenia. Psychiatry Research: Neuroimaging, 234(1), 35–43. 10.1016/j.pscychresns.2015.08.003 26341951

[r71] Poletti, S., Vai, B., Smeraldi, E., Cavallaro, R., Colombo, C., & Benedetti, F. (2016). Adverse childhood experiences influence the detrimental effect of bipolar disorder and schizophrenia on cortico-limbic grey matter volumes. Journal of Affective Disorders, 189, 290–297. 26454335 10.1016/j.jad.2015.09.049

[r72] Quidé, Y., O’Reilly, N., Rowland, J. E., Carr, V. J., Elzinga, B. M., & Green, M. J. (2017a). Effects of childhood trauma on working memory in affective and non-affective psychotic disorders. Brain Imaging and Behavior, 11(3), 722–735. 27090803 10.1007/s11682-016-9548-z

[r73] Quidé, Y., O’Reilly, N., Watkeys, O. J., Carr, V. J., & Green, M. J. (2018). Effects of childhood trauma on left inferior frontal gyrus function during response inhibition across psychotic disorders. Psychological Medicine, 48(9), 1454–1463. 28994360 10.1017/S0033291717002884

[r74] Quidé, Y., Ong, X. H., Mohnke, S., Schnell, K., Walter, H., Carr, V. J., & Green, M. J. (2017b). Childhood trauma-related alterations in brain function during a theory-of-mind task in schizophrenia. Schizophrenia Research, 189, 162–168. 28215391 10.1016/j.schres.2017.02.012

[r75] Quidé, Y., Girshkin, L., Watkeys, O. J., Carr, V. J., & Green, M. J. (2021). The relationship between cortisol reactivity and emotional brain function is differently moderated by childhood trauma, in bipolar disorder, schizophrenia and healthy individuals. European Archives of Psychiatry and Clinical Neuroscience, 271(6), 1089–1109. 32926285 10.1007/s00406-020-01190-3

[r76] Quidé, Y., Watkeys, O. J., Tonini, E., Grotegerd, D., Dannlowski, U., Nenadić, I., … Green, M. J. (2024). Childhood trauma moderates schizotypy-related brain morphology: Analyses of 1182 healthy individuals from the ENIGMA schizotypy working group. Psychological Medicine, 54(6), 1215–1227. 37859592 10.1017/S0033291723003045

[r79] Read, J., & Argyle, N. (1999). Hallucinations, delusions, and thought disorder among adult psychiatric inpatients with a history of child abuse. Psychiatric Services, 50(11), 1467–1472. 10543857 10.1176/ps.50.11.1467

[r80] Read, J., Fosse, R., Moskowitz, A., & Perry, B. (2014). The traumagenic neurodevelopmental model of psychosis revisited. Neuropsychiatry, 4(1), 65–79.

[r81] Ruby, E., Rothman, K., Corcoran, C., Goetz, R. R., & Malaspina, D. (2017). Influence of early trauma on features of schizophrenia. Early Intervention in Psychiatry, 11(4), 322–333. 25808607 10.1111/eip.12239 PMC4580512

[r82] Sapolsky, R. M. (1996). Stress, glucocorticoids, and damage to the nervous system: The current state of confusion. Stress, 1(1), 1–19. 9807058 10.3109/10253899609001092

[r83] Schäfer, I., & Fisher, H. L. (2011). Childhood trauma and psychosis-what is the evidence? Dialogues in Clinical Neuroscience, 13(3), 360–365. 22033827 10.31887/DCNS.2011.13.2/ischaefer PMC3182006

[r84] Sheffield, J. M., Williams, L. E., Woodward, N. D., & Heckers, S. (2013). Reduced gray matter volume in psychotic disorder patients with a history of childhood sexual abuse. Schizophrenia Research, 143(1), 185–191. 23178105 10.1016/j.schres.2012.10.032 PMC3540174

[r86] Stevens, L. H., Spencer, H. M., & Turkington, D. (2017). Identifying four subgroups of trauma in psychosis: Vulnerability, psychopathology, and treatment. Frontiers in Psychiatry, 8, 21. 28243211 10.3389/fpsyt.2017.00021 PMC5303718

[r87] Teicher, M. H., Anderson, C. M., Ohashi, K., Khan, A., McGreenery, C. E., Bolger, E. A., … Vitaliano, G. D. (2018). Differential effects of childhood neglect and abuse during sensitive exposure periods on male and female hippocampus. NeuroImage, 169, 443–452. 29288867 10.1016/j.neuroimage.2017.12.055 PMC5856615

[r88] Teicher, M. H., & Samson, J. A. (2016). Annual research review: Enduring neurobiological effects of childhood abuse and neglect. Journal of Child Psychology and Psychiatry, 57(3), 241–266. 26831814 10.1111/jcpp.12507 PMC4760853

[r89] Teicher, M. H., Samson, J. A., Anderson, C. M., & Ohashi, K. (2016). The effects of childhood maltreatment on brain structure, function and connectivity. Nature Reviews Neuroscience, 17(10), 652–666. 27640984 10.1038/nrn.2016.111

[r90] Thomas, M., Rakesh, D., Whittle, S., Sheridan, M., Upthegrove, R., & Cropley, V. (2023). The neural, stress hormone and inflammatory correlates of childhood deprivation and threat in psychosis: A systematic review. Psychoneuroendocrinology, 106371. 37651860 10.1016/j.psyneuen.2023.106371

[r91] Üçok, A., Kaya, H., Uğurpala, C., Çıkrıkçılı, U., Ergül, C., Yokuşoğlu, Ç., … Direk, N. (2015). History of childhood physical trauma is related to cognitive decline in individuals with ultra-high risk for psychosis. Schizophrenia Research, 169(1–3), 199–203. 26386899 10.1016/j.schres.2015.08.038

[r92] Varese, F., Smeets, F., Drukker, M., Lieverse, R., Lataster, T., Viechtbauer, W., … Bentall, R. P. (2012). Childhood adversities increase the risk of psychosis: A meta-analysis of patient-control, prospective-and cross-sectional cohort studies. Schizophrenia Bulletin, 38(4), 661–671. 22461484 10.1093/schbul/sbs050 PMC3406538

[r93] Wells, G. A., Shea, B., O’Connell, D., Peterson, J., Welch, V., Losos, M., & Tugwell, P. (2011). The Newcastle-Ottawa scale (NOS) for assessing the quality of nonrandomized studies in meta-analysis. Ottawa Hospital Research Institute. http://www.ohri.ca/programs/clinical_epidemiology/oxford.asp.

[r94] Xie, M., Cai, J., Liu, Y., Wei, W., Zhao, Z., Dai, M., … Li, T. (2023). Association between childhood trauma and white matter deficits in first-episode schizophrenia. Psychiatry Research, 323, 115111. 36924585 10.1016/j.psychres.2023.115111

[r95] Zammit, S., Lewis, C., Dawson, S., Colley, H., McCann, H., Piekarski, A., … Bisson, J. (2018). Undetected post-traumatic stress disorder in secondary-care mental health services: Systematic review. The British Journal of Psychiatry, 212(1), 11–18. 29433609 10.1192/bjp.2017.8 PMC6457163

